# Sorting nexin 17 increases low-density lipoprotein receptor-related protein 4 membrane expression: A novel mechanism of acetylcholine receptor aggregation in myasthenia gravis

**DOI:** 10.3389/fimmu.2022.916098

**Published:** 2022-10-12

**Authors:** Xiaoxiao He, Shuxian Zhou, Ying Ji, Yingna Zhang, Jie Lv, Shangkun Quan, Jing Zhang, Xue Zhao, Weike Cui, Wenbo Li, Peipei Liu, Linyuan Zhang, Tong Shen, Hua Fang, Junhong Yang, Yunke Zhang, Xinzheng Cui, Qingyong Zhang, Feng Gao

**Affiliations:** ^1^ BGI College, Zhengzhou University, Zhengzhou, China; ^2^ Department of Neuroimmunology, Henan Institute of Medical and Pharmaceutical Sciences, Zhengzhou University, Zhengzhou, China; ^3^ Basic Medical College, Zhengzhou University, Zhengzhou, China; ^4^ Department of Neurology, The Second Affiliated Hospital of Zhengzhou University, Zhengzhou, China; ^5^ Department of Pathophysiology, School of Basic Medical Sciences, Ningxia Medical University, Yinchuan, China; ^6^ Department of Encephalopathy, The First Affiliated Hospital of Henan University of Chinese Medicine, Zhengzhou, China; ^7^ Myasthenia Gravis Comprehensive Diagnosis and Treatment Center, Henan Provincial People’s Hospital, Zhengzhou, China

**Keywords:** sorting nexin 17, low-density lipoprotein receptor-related protein 4, acetylcholine receptor, myasthenia gravis, endplate membranes

## Abstract

Myasthenia gravis (MG) is characterized by autoimmune damage to the postsynaptic membrane of the neuromuscular junction (NMJ) with impaired postsynaptic acetylcholine receptor (AChR) aggregation. Low-density lipoprotein receptor-related protein 4 (LRP4) plays an important role in AChR aggregation at endplate membranes *via* the Agrin–LRP4–muscle-specific receptor tyrosine kinase (MuSK) cascade. Sorting nexin 17 (SNX17) regulates the degradation and recycling of various internalized membrane proteins. However, whether SNX17 regulates LRP4 remains unclear. Therefore, we examined the regulatory effects of SNX17 on LRP4 and its influence on AChR aggregation in MG. We selected C2C12 myotubes and induced LRP4 internalization *via* stimulation with anti-LRP4 antibody and confirmed intracellular interaction between SNX17 and LRP4. SNX17 knockdown and overexpression confirmed that SNX17 promoted MuSK phosphorylation and AChR aggregation by increasing cell surface LRP4 expression. By establishing experimental autoimmune MG (EAMG) mouse models, we identified that SNX17 upregulation improved fragmentation of the AChR structure at the NMJ and alleviated leg weakness in EAMG mice. Thus, these results reveal that SNX17 may be a novel target for future MG therapy.

## 1 Introduction

Myasthenia gravis (MG) is a typical autoimmune disease induced by autoantibodies. The pathological feature is autoimmune damage to the postsynaptic membrane of the neuromuscular junction (NMJ), leading to impaired acetylcholine receptor (AChR) aggregation at the postsynaptic membrane (1). Promoting normal aggregation of AChR in MG enables better binding to the acetylcholine (ACh) released by the presynaptic membrane, ensuring normal transmission of excitatory signals from the NMJ and aiding recovery of muscle contraction function in MG patients (2, 3). Studies have shown that the Agrin–LRP4–MuSK signaling cascade, comprising Agrin, low-density lipoprotein receptor (LDLR)-related protein 4 (LRP4), and muscle-specific receptor tyrosine kinase (MuSK), plays an important role in neuromuscular excitation transmission by affecting the density and function of AChR. Thus, this cascade may be an important candidate for the treatment of MG and other NMJ diseases (4, 5).

LRP4 acts as a type I single-pass transmembrane protein and an important component of the endplate membrane signaling pathway. In 2013, LRP4 autoantibodies were found to induce LRP4 internalization by crosslinking with LRP4 resulting in reduced LRP4 cell surface expression and affecting AChR aggregation through the Agrin–LRP4–MuSK signaling pathway. This is an important mechanism for anti-LRP4 antibody-positive MG (LRP4-MG) (6). Therefore, maintaining the stability of LRP4 on the muscle cell membrane is crucial for ensuring normal AChR aggregation and neuromuscular excitation transmission (7).

Sorting nexin 17 (SNX17) is a member of the sorting nexin family with a typical phox-homology (PX) domain that localizes SNX17 to intracellular vesicles by binding to specific phosphatidyl inositols (8, 9). In addition to the PX domain, SNX17 possesses a FERM domain that can accurately identify internalized membrane proteins harboring NPxY/NxxY motifs and regulate the recycling and degradation of internalized membrane proteins (10, 11). Studies suggest that the majority of the LDLR family located on various cell membranes contain the NPxY/NxxY motifs in their intracellular regions (e.g., LRP1 and ApoER2). SNX17 binds to the intracellular region of LDLR through the FERM domain, which prevents it from entering lysosomes for degradation after endocytosis, thereby promoting recycling to the membrane to re-perform receptor function (12–14).

LRP4, a member of the LDLR protein family, has a protein structure and topological characteristics similar to that of other members. It also contains a NPxY motif in its intracellular domain (15). This suggests that SNX17 may affect AChR aggregation by regulating LRP4. Therefore, this study focused on elucidating the relationship between SNX17 and LRP4 and its effect on AChR aggregation.

## 2 Materials and methods

### 2.1 C2C12 cell culture and treatment

The C2C12 mouse myoblast cell line was purchased from Cell Bank of the Chinese Academy of Sciences (Shanghai, China), was cultured in DMEM (HyClone, UT, USA) containing 10% fetal bovine serum (FBS, Tianhang Biotechnology Co., Ltd., Zhejiang, China), and maintained in an incubator at 37°C and 5% CO_2_. When the cell growth density was approximately 70%, the medium was replaced with DMEM containing 2% horse serum (Gibco, NY, USA) to induce differentiation. During this period, the medium was changed daily to induce differentiation of C2C12 cells into myotubes.

### 2.2 Lentiviral transduction in C2C12 cells

C2C12 cells were seeded in six-well plates at 3×10^5^/mL cell density. When these cells reached 30%–50% density, 1 mL of DMEM containing 3 × 10^7^ TU/mL of lentiviruses expressing SNX17 knockdown and overexpressed sequences (LV-m-SNX17 shRNA-ZsGreen-PURO and LV-m-SNX17-3xflag-ZsGreen-PURO) and 10% FBS was added. When the cells were transduced with lentiviruses for 4 h, 1 mL of DMEM containing 10% FBS was added. Furthermore, DMEM containing lentiviruses was replaced 24 h after transduction. Thereafter, the cells were placed in an incubator for 48 h. The lentiviruses were synthesized by Hanbio Co., Ltd. (Shanghai, China).

### 2.3 Establishment and grouping of experimental autoimmune MG models

**Figure 1:**
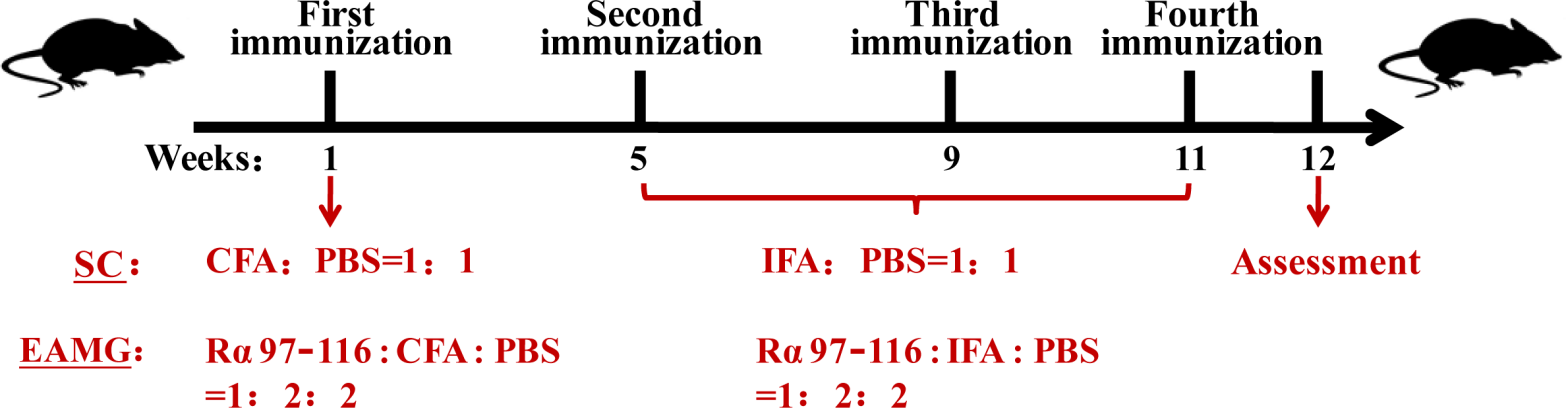
Animal modeling process. After 1 week of adaptive feeding, 51 female C56BL/6J mice were randomly divided into the normal control (NC) (*n* = 6), solvent control (SC) (*n* = 5), and experimental autoimmune myasthenia gravis (EAMG) (*n* = 40) groups. The mice in the SC group and the EAMG group received the first immunization in the first week, the second immunization in the fifth week, the third immunization in the ninth week, and the fourth immunization in the eleventh week. The EAMG model was assessed in the twelfth week.

Fifty-one healthy, specific pathogen-free (SPF), C57BL/6J female mice (6-8-week-old, weighing 16-18 g) were purchased from SJA Laboratory Animal Co., Ltd. (Hunan, China). As MG has a higher prevalence in women, female mice were selected for this animal model. The animals were reared at the SPF Animal Center of the Henan Institute of Medical and Pharmaceutical Sciences, with 45% humidity, 22–25°C temperature, 12 h light-dark, and good ventilation. One week after adaptive feeding and observation, the mice were randomly divided into normal control (NC) (*n* = 6), solvent control (SC) (*n* = 5), and experimental autoimmune MG (EAMG) (*n* = 40) groups. Mice in the NC group were fed normally without treatment. However, mice in the EAMG group were treated as described below with reference to the description of Jing et al. (16). The Rα 97–116 peptide of the AChR-α subunit (synthesized by Shanghai Top-Peptide Biotechnology Co., Ltd., China) was selected as the immunogen. Immunization was performed according to the timeline shown in **Figure 1**. In the first immunization, Rα 97–116, complete Freund’s adjuvant (CFA, Sigma, NJ, USA), and phosphate-buffered saline (PBS, Solarbio, Beijing, China) were thoroughly mixed at a ratio of 1:2:2 to prepare an immune emulsion. The emulsion (200 μL, containing 50 μg of Rα 97–116) was divided into four parts and injected into the both hind footpads and subcutaneous scapula of mice in the EAMG group. Mice in the SC group were simultaneously injected with an emulsion containing only CFA and PBS in a 1:1 ratio without Rα 97-116 according to the injection protocol for EAMG mice as solvent controls. However, CFA in the emulsion injected during the second, third, and fourth immunizations was replaced with incomplete Freund’s adjuvant (IFA, Sigma, NJ, USA). The EAMG model was assessed one week after the fourth immunization. This study was approved by the Laboratory Animal Management and Ethics Committee of the Henan Institute of Medical and Pharmaceutical Sciences and followed the animal welfare regulations.

### 2.4 Evaluation of EAMG model

To evaluate the EAMG mouse model, we included a total of four evaluation methods. 1) Clinical scores (17): Used to evaluate disease characteristics in mice and scored from low to high based on severity of muscle weakness symptoms. Due to the subjective nature of the clinical score, it is usually necessary to use it in combination with the flip suspension test to evaluate the muscle strength of the mouse model as a whole. 2) Neostigmine test: This is an important pharmacological experiment for clinical diagnosis of MG. Signaling between NMJ synapses is enhanced by increasing the amount of ACh in the muscle by injecting the cholinesterase inhibitor drug neostigmine into the muscle. Patients with MG usually experience significant relief of symptoms of muscle weakness 20 min after injection. However, concurrent injection of atropine is required to counteract the muscarinic reaction of neostigmine. 3) Detection of AChR-Ab in mouse serum. 4) Electromyography (EMG) changes in mouse gastrocnemius muscles were detected using low-frequency repetitive nerve stimulation (RNS).

The criteria for evaluating the success of the EAMG model were as follows: 1) clinical scores ≥1 point; 2) a positive neostigmine test; 3) positive AChR-Ab in the serum; and 4) attenuation of low-frequency RNS potential >10%. If criteria 1) along with 2), 3), or 4) were present, then the EAMG model was considered to have been successfully prepared.

#### 2.4.1 Measurement of muscle strength in laboratory animals

The clinical scores were obtained in 51 mice 1 week after the fourth immunization, and the standard of the evaluation was based on Lennon’s (18) grading method, which divides the severity of symptoms into five grades. Grade 0: normal muscle strength, no signs of fatigue; Grade 1: weak bite, decreased activity, and easy fatigue; Grade 2: obvious weakness, protruding body posture at rest, dropping head and tail, bent in the forelimb toes, and unsteady walking; Grade 3: severe weakness, no biting action, unsupported legs, and difficulty breathing; and Grade 4: death. Based on these symptoms, grades 0, 1, 2, 3, and 4 were assigned 0, 1, 2, 3, and 4 points, respectively. Symptoms in between each grade were assigned 0.5, 1.5, and 2.5 points, respectively.

Owing to the strong subjectivity of the clinical scores, when measuring the muscle strength of experimental animals, the degree of fatigue was evaluated using the flip suspension test, during which the experimental animals are forced to completely grasp an iron net with their limbs and then slowly turn the iron net such that the entire body weight is completely supported on the limbs. The sum of the time spent by the mouse for two consecutive drops was recorded. If the hanging time was shorter than 10 s, it is not recorded, and the measurement was performed again.

#### 2.4.2 Neostigmine test (19)

One (19) week after the fourth immunization, 10 model mice were randomly selected for intraperitoneal injection with 37.5 μg/kg neostigmine (Xinyi Pharmaceutical Co., Ltd., Shanghai, China) and 15 μg/kg atropine (Suicheng Pharmaceutical Co., Ltd., Henan, China), and the symptoms of muscle weakness in the mice were observed after 20 min. If activity increased, muscle strength during grasping significantly improved, and this lasted for >2 h, it was deemed as a positive neostigmine test.

#### 2.4.3 Detection of AChR-Ab in serum

One week after the fourth immunization, blood was collected by tail clipping from 51 mice, and the blood was left to stand for 30 min, centrifuged at 2,000 rpm for 10 min at 4°C, and the upper serum was collected. Rα 97–116 peptides were dissolved in carbonate buffer and coated on 96-well plates at 0.05 μg/well. Twelve hours after the blocking step with 5% BSA (Genview, USA), mouse serum from each group was added to 96-well plates at 1:10 dilution with PBS and incubated at 37°C for 1 h. The 96-well plates were washed five times with 0.05% PBST, and then, 1:6,000-diluted HRP-goat anti-mouse IgG (IH-0031, Dingguochangsheng Co., Ltd., Beijing, China) was added to each well and incubated at 37°C for 1 h. After washing the 96-well plates five times with 0.05% PBST again, TMB chromogenic reagent was added to the plates and incubated at 37°C for 30 min away from light. Finally, 2 M H_2_SO_4_ solution was added to stop the reaction. Absorbance of serum samples from each group was measured at 450 nm using TECAN multiplate reader. AChR-Ab detection used P/N as the evaluation index, where P/N = (sample absorbance - blank absorbance)/(control absorbance - blank absorbance). The average value of absorbance of the mouse serum in the NC group was used as control absorbance: if P/N was greater than 2.1, AChR-Ab was determined as positive.

#### 2.4.4 Low-frequency repetitive nerve stimulation

Based on the method by Jiang et al. (20), after anesthetizing the mice with an intraperitoneal injection of 10% chloral hydrate (Kemiou Chemical Reagent Co., Ltd., Tianjin, China) and fixing them on a fixation plate with tape, a stimulating electrode was inserted into the muscle tissue near the sciatic nerve, a reference electrode was inserted into the base of the tail, and a recording electrode was inserted into the gastrocnemius muscle tissue. A BIOPAC MP150 multichannel physiological signal recorder was used to record changes in EMG of the gastrocnemius muscle of mice for 5 consecutive electrical stimulations at 3 Hz. Then, the attenuation rate of the fifth amplitude of EMG compared with the first one was calculated. The attenuation rate (D) was calculated as: D (%) = (action potential 1 - action potential 5)/action potential 1 × 100%. D (%) ≥10% was considered positive.

### 2.5 Establishment of SNX17 knockdown and overexpression EAMG models

Twenty-six mice were evaluated to meet the EAMG standard; the success rate of the model was 65%. Twenty-six EAMG mice were randomly divided into four groups: *SNX17* knockdown control (SNX17 KD-con, *n* = 5), *SNX17* knockdown (SNX17 KD, *n* = 8), *SNX17* overexpression control (SNX17 OE-con, *n* = 5), and *SNX17* overexpression (SNX17 OE, *n* = 8). Adeno-associated virus with AAV2/9-EGFP NC, AAV2/9-m-SNX17 shRNA-EGFP, AAV2/9-ZsGreen, AAV2/9-CMV-m-SNX17-3xflag-ZsGreen (Hanbio Co., Ltd., Shanghai, China) constructs were sequentially injected into the gastrocnemius muscle of the right leg of mice in each group at five points (20 μL/point). The left leg of mice in each group was used as the model control. Twenty-one days post-injection, mice were gas anesthetized (1%–1.5% isoflurane in the air) and placed in an observation box. *In vivo* molecular imaging of mice injected with EGFP-tagged adeno-associated virus was performed using the IVIS Lumina III (PerkinElmer, MA, USA) Small Animal Intravital Optical Imaging System to observe viral infection in the right leg of the mice. Images were captured using Living image software at 20°C, and ROI regions of the images were quantitatively analyzed. Images were cropped and resized using Adobe Photoshop after acquisition.

### 2.6 Western blotting

Lentivirus-infected C2C12 myotubes were treated with 10 μg/mL of LRP4 antibody (MA5-27675, Thermo Fisher Scientific, MA, USA) for 30 min, after which the antibody was removed. The cells were washed three times with PBS, suspended in DMEM containing 10% FBS, and placed in an incubator at 37°C and 5% CO_2_ for 30 min, and then the membrane proteins were obtained using a membrane protein extraction kit (Beyotime, Shanghai, China). Following the same treatment method, the cells were incubated in DMEM containing 10 ng/mL of Agrin (550-AG-100, R&D Systems, MN, USA) for 16 h after removing the LRP4 antibody. Cultured cells or tissue samples were then lysed using pre-chilled RIPA lysis buffer (Solarbio, Beijing, China) containing 1 mM PMSF (Solarbio, Beijing, China) and centrifuged at 12,000 rpm for 15 min to obtain total cellular protein. The concentration of the collected proteins was determined using the BCA Protein Assay kit (Beyotime, Shanghai, China), and an equal amount of protein sample was loaded onto a polyacrylamide gel and separated by SDS-PAGE. The proteins were then transferred to PVDF membranes (Merck Millipore, MA, USA), blocked with 5% nonfat dry milk (Dingguochangsheng Co., Ltd., Beijing, China), and incubated with primary and secondary antibodies. Primary antibodies included anti-SNX17 (1:1,000, 10275-1-AP, Proteintech, IL, USA), anti-LRP4 (1:1,000), anti-MuSK (1:500, sc-517346, Santa Cruz Biotechnology, CA, USA), anti-phospho-MuSK (Tyr755) (1:2,000, AF7108, Affinity, OH, USA), anti-GAPDH (1:1,000, GB12002, Servicebiio, Wuhan, China), anti-tubulin (1:500, sc-166729, Santa Cruz Biotechnology, CA, USA), and anti-Na/K ATPase (1:20,000, ab76020, Abcam, Cambridge, UK). The secondary antibodies included HRP-goat anti-mouse IgG and HRP-goat anti-rabbit IgG (both 1:8,000, IH-0011, Dingguochangsheng Co., Ltd., Beijing, China). The protein bands were identified using the Enhanced Pico Light Chemiluminescence Kit (Epizyme, Shanghai, China) according to the manufacturer’s instructions. Relative protein expression levels were analyzed using ImageJ software.

### 2.7 Quantitative real-time polymerase chain reaction

Total RNA was extracted from tissue samples or cultured cells using an RNA isolation kit (Tiangen Biochemical Co., Ltd., Beijing, China), according to the manufacturer’s protocol. RNA samples were quantified, and cDNA was synthesized using reverse transcription kit HiScript III-RT SuperMix for q-PCR (Vazyme, Nanjing, China). cDNA was amplified by PCR in a real-time cycler using ChamQ Universal SYBR qPCR Master Mix (Vazyme, Nanjing, China). All experiments were performed in triplicates, and the data were normalized against the expression of *GAPDH* as a housekeeping gene. The primer sequences of SNX17 (forward: 5′ GGTGTCAGACATTGAGCATGGC 3′; reverse: 5′ AGCCATAGTGCCGCAGAGTTTG 3′) and GAPDH (forward: 5′ CATCACTGCCACCCAGAAGACTG 3′; reverse: 5′ ATGCCAGTGAGCTTCCCGTTCAG 3′) were both synthesized by Sangon Bioengineering Co., Ltd. (Shanghai, China).

### 2.8 Co-immunoprecipitation

After C2C12 cells were treated with 10 μg/mL of LRP4 antibody for 30 min, they were lysed with binding buffer containing 50 mM Tris, 100 mM NaCl, 0.5% Triton X-100, pH 7.5, and 1 mM PMSF on ice for 15 min. Thereafter, the cells were centrifuged at 12,000 rpm for 15 min to obtain protein solution. Anti-SNX17 antibody, anti-LRP4 antibody, and anti-mouse IgG antibody (ab190475, Abcam, Cambridge, UK) were diluted with the same binding buffer to 20 μg/mL and incubated with protein A/G immunoprecipitation magnetic beads (Bimake, TX, USA) at 4°C for 4 h. A protein solution was added to the magnetic bead-antibody mixture and incubated at 4°C for 12 h. Samples were then washed three times with the same binding buffer. Proteins were eluted from the magnetic beads by boiling in the presence of 5X SDS-PAGE loading buffer (Beyotime, Shanghai, China) for 10 min. Eluted proteins were then subjected to western blotting analysis.

### 2.9 Immunofluorescence

Lentivirus-infected C2C12 myotubes were treated with LRP4 antibody and Agrin, as described in the Western Blotting subsection, fixed with 4% paraformaldehyde (Leagene, Beijing, China) for 15 min at 37°C, and permeabilized with 0.05% Triton X-100 (TCI, Tokyo, Japan) for 20 min. The cells were blocked with 5% BSA for 2 h at 37°C and incubated with rhodamine-labeled bungarotoxin (R-BTX, Sigma, NJ, USA) at 1:500 dilution for 12 h at 4°C. The next day, the cells were stained with DAPI (Servicebio, Wuhan, China) for 8 min and washed three times with PBS for 10 min each. Images were captured using Nikon TS100-F (40× oil objective, NA = 1.40) inverted fluorescence microscope at 18°C. The number of AChR clusters formed by AChR aggregation on myotubes in each field was counted, and the length of individual AChR clusters was analyzed using the straight line tool in ImageJ software. Fresh mouse gastrocnemius muscles were fixed in 4% paraformaldehyde for 30 min, and the myofilaments were then stripped under a microscope. The stripped myofilaments were stained with R-BTX as described above and images were captured using Olympus FA1000 confocal microscope at 18°C. All images were captured without gamma adjustment. After capture, use Adobe Photoshop to fine-tune the brightness and contrast of the image, then rotate crop, and resize. And composited using Adobe Illustrator. Images were analyzed using ImageJ software.

### 2.10 Hematoxylin-eosin staining

The gastrocnemius muscles of the mice in each group were embedded in wax and cut into 4–8 μm slices according to the direction of the muscle fibers. After hydration, the slides were stained with hematoxylin using the hematoxylin-eosin (HE) staining kit (Leagene, Beijing, China) for 5 min and placed in running water until the nuclei turned blue and then stained with eosin for 3 min. The slides were dehydrated and sealed with neutral gum, and images were captured using an Olympus BX53 light microscope at 18°C.

### 2.11 Measurement of the unilateral leg strength in mice

After the *SNX17* knockdown and overexpression EAMG models were successfully established, the force measuring rod of the 1N bar dynamometer was connected to the right foot of the mice. When the positions of the bar dynamometer and the mouse were fixed, a certain stimulation was applied to the tested leg of the mouse, and the maximum force reached by the mouse pulling the dynamometer rod was recorded (unit: N).

### 2.12 Statistical analysis

Data are expressed as mean ± SEM. The *t*-test was used to compare the means between two independent samples, and one-way ANOVA was used to compare the means of multiple groups of samples. *P*<0.05 was considered to be statistically significant.

## 3 Results

### 3.1 SNX17 knockdown decreased cell surface LRP4 expression, MuSK phosphorylation, and AChR aggregation

**Figure 2:**
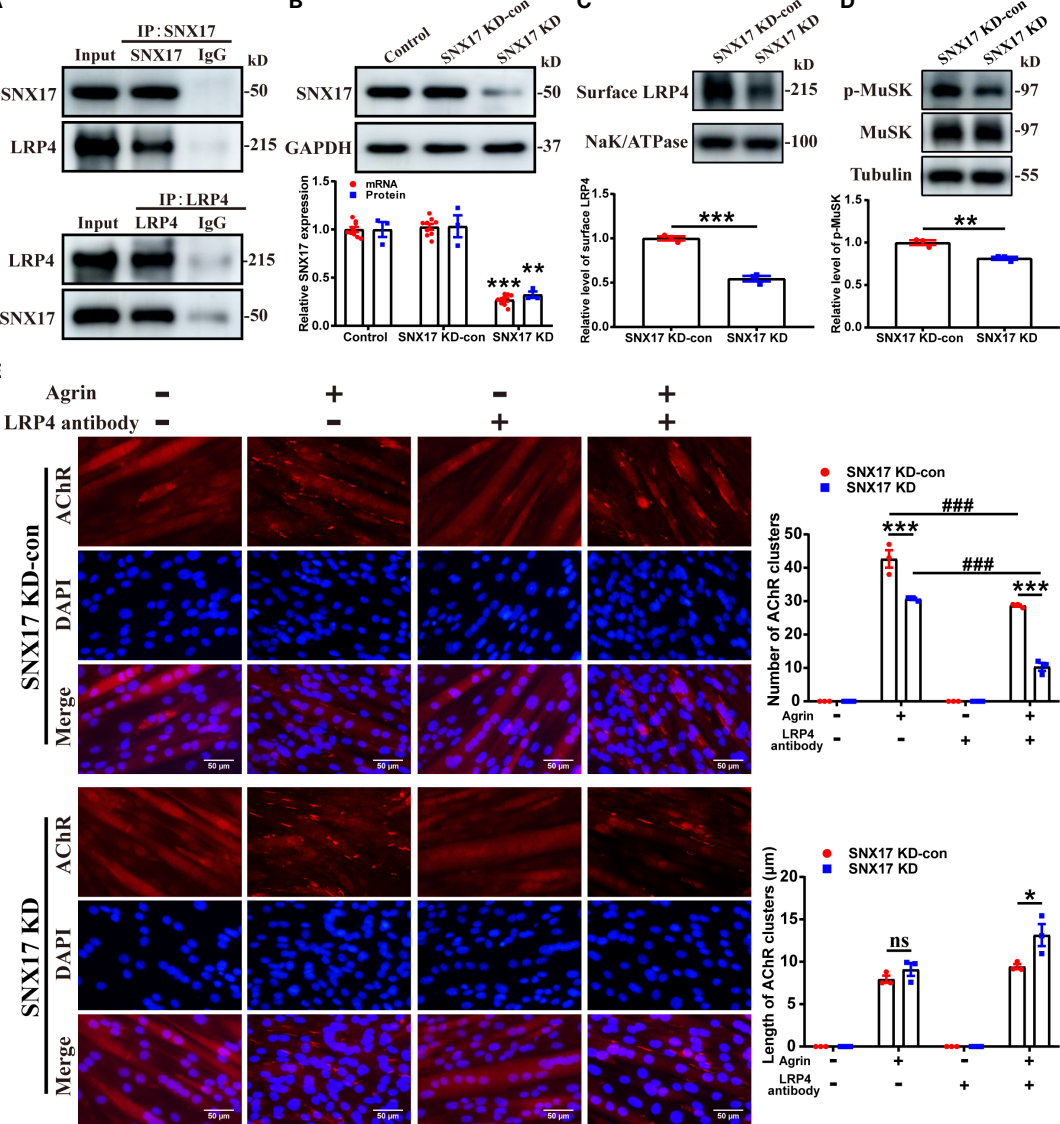
SNX17 knockdown decreased cell surface LRP4 expression, MuSK phosphorylation, and AChR aggregation. **(A)** Cell lysates were immunoprecipitated with antibodies against SNX17, LRP4, or control IgG. The LRP4 and SNX17 proteins in immunoprecipitation and input were analyzed by western blotting. **(B)** Western blotting and qPCR detection of SNX17 protein (n = 3/group) and mRNA (n = 9/group) expression, respectively, in lentivirus-infected cells. **(C)** Myotube cells were treated with 10 μg/mL anti-LRP4 antibody for 30 min to induce internalization of LRP4 protein on the membrane, following which the anti-LRP4 antibody was removed and the cells were cultured for 30 min, and then the content of LRP4 protein in the membrane protein was analyzed by Western Blotting. n = 3/group. **(D)** Myotube cells were treated with 10 μg/mL anti-LRP4 antibody for 30 min, after which the anti-LRP4 antibody was removed, the cells were incubated with 10 ng/mL Agrin for 16 h, and the expression of MuSK and p-MuSK in total protein was analyzed by Western Blotting. n = 3/group. **(E)** AChR clusters (red) on lentivirus-infected C2C12 myotubes were labeled with R-BTX after application of separate or mixed Agrin and anti-LRP4 antibody, the nuclei were stained with DAPI (blue), and the number of AChR clusters on myotubes was counted and the length of AChR clusters was analyzed by ImageJ software. n = 3/group; Scale bar = 50 μm. * represents SNX17 KD-con vs. SNX17 KD, # represents -LRP4 antibody vs. +LRP4 antibody. All data are presented as mean ± SEM. *P<0.05, **P<0.01, ***P<0.001, ^###^ *P*<0.001. AChR, acetylcholine receptor; EAMG, experimental autoimmune myasthenia gravis; LRP4, low-density lipoprotein receptor-related protein 4; MuSK, muscle-specific receptor tyrosine kinase; R-BTX, rhodamine-labeled bungarotoxin; SEM, Standard Error of Mean; SNX17, sorting nexin 17. ns, no significance.

First, we verified the endogenous interaction between SNX17 and LRP4 by co-immunoprecipitation. A direct interaction between SNX17 and LRP4 was identified, regardless of whether SNX17 or LRP4 was precipitated (**Figure 2A**). Next, to investigate the effect of SNX17 on cell surface LRP4 expression after induction of cell surface LRP4 internalization, we infected C2C12 myotubes with lentiviral shRNA targeting *SNX17* for SNX17 knockdown (**Figure 2B**). After SNX17 had been knocked down in myotubes, we used 10 μg/mL anti-LRP4 antibody to induce internalization of LRP4 protein on the cell surface, then removed the anti-LRP4 antibody and continued to culture for 30 min to detect the content of LRP4 protein in membrane proteins by Western Blotting. However, *SNX17* knockdown significantly reduced cell surface LRP4 expression (0.55 ± 0.03) (**Figure 2C**) and decreased the phosphorylation levels of MuSK (0.82 ± 0.02) more than that in the SNX17 KD-con group after Agrin stimulation (**Figure 2D**). Furthermore, We also explored the effects of SNX17 on AChR aggregation. After treating C2C12 myotube cells with a separate or mixed application of Agrin and anti-LRP4 antibody, the number and length of AChR clusters formed by AChR aggregation on myotubes were statistically analyzed. We found that AChR aggregation in C2C12 myotubes was strictly dependent on the presence of Agrin. Agrin-alone application in the SNX17 KD group slightly reduced the number of AChR clusters (30.67 ± 0.33) compared to the SNX17 KD-con group (42.67 ± 2.60). By contrast, the number of AChR clusters in the SNX17 KD-con group (28.67 ± 0.33) was only slightly decreased after application of mixed Agrin and anti-LRP4 antibody treatment, while the number of AChR clusters in the SNX17 KD group (10.33 ± 1.20) was significantly reduced. However, the length of AChR clusters in the SNX17 KD group (13.15 ± 1.29 μm) was slightly longer than that in the SNX17 KD-con group (9.43 ± 0.30 μm) (**Figure 2E**). Although it is unclear why knockdown of SNX17 resulted in a slight increase in the length of AChR clusters, knockdown of SNX17 significantly reduced the number of AChR clusters formed by AChR aggregation. These results demonstrate the inhibitory effect of SNX17 knockdown on AChR aggregation in myotubes.

### *3.2 SNX17* overexpression increased cell surface LRP4 expression, MuSK phosphorylation, and AChR aggregation

**Figure 3:**
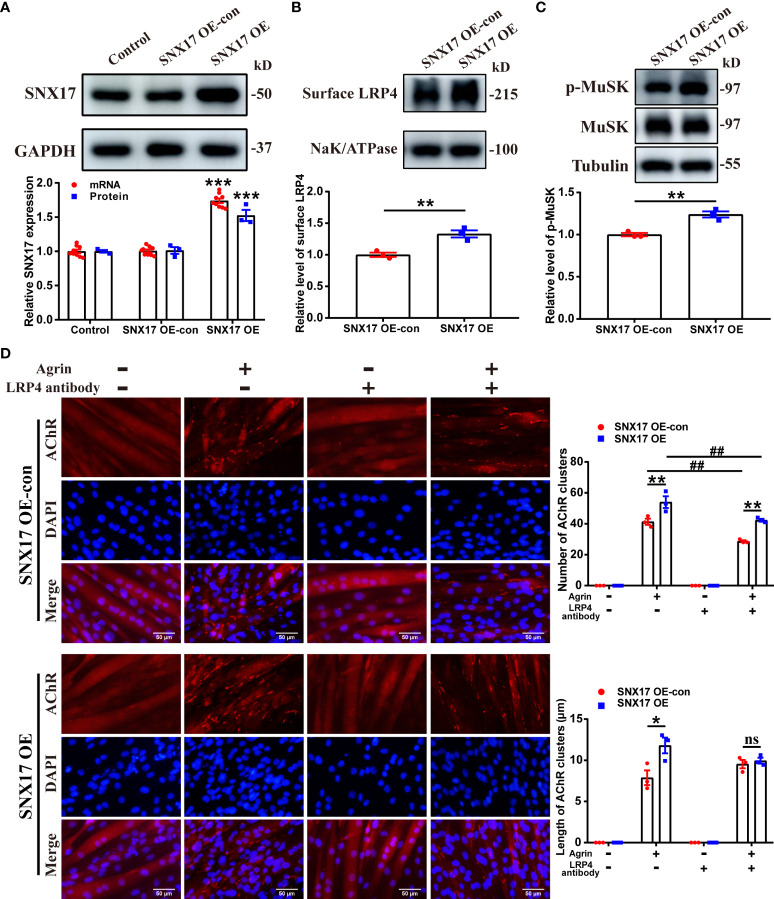
SNX17 overexpression increased cell surface LRP4 expression, MuSK phosphorylation, and AChR aggregation. **(A)** Western blotting and qPCR detection of SNX17 protein (n = 3/group) and mRNA (n = 9/group) expression, respectively, in lentivirus-infected cells. **(B)** Myotube cells were treated with 10 μg/mL anti-LRP4 antibody for 30 min to induce internalization of LRP4 protein on the membrane, following which the anti-LRP4 antibody was removed and the cells were cultured for 30 min, and then the content of LRP4 protein in the membrane protein was analyzed by Western Blotting. n = 3/group. **(C)** Myotube cells were treated with 10 μg/mL anti-LRP4 antibody for 30 min, after which the anti-LRP4 antibody was removed, the cells were incubated with 10 ng/mL Agrin for 16 h, and the expression of MuSK and p-MuSK in total protein was analyzed by Western Blotting. n = 3/group. **(D)** AChR clusters (red) on lentivirus-infected C2C12 myotubes were labeled with R-BTX after application of separate or mixed Agrin and anti-LRP4 antibody, the nuclei were stained with DAPI (blue), and the number of AChR clusters on myotubes was counted and the length of AChR clusters was analyzed by ImageJ software. n = 3/group; Scale bar = 50 μm. * represents SNX17 OE-con vs. SNX17 OE. All data are presented as mean ± SEM. *P<0.05, **P<0.01, ***P<0.001, ^##^ *P*<0.01. AChR, acetylcholine receptor; EAMG, experimental autoimmune myasthenia gravis; LRP4, low-density lipoprotein receptor-related protein 4; MuSK, muscle-specific receptor tyrosine kinase; R-BTX, rhodamine-labeled bungarotoxin; SEM, Standard Error of Mean; SNX17, sorting nexin 17. ns, no significance.

Next, we developed myotubes overexpressing SNX17 (**Figure 3A**). Consistent with the previously described treatment of SNX17-knockdown myotubes, we also examined LRP4 protein content in SNX17-overexpressing myotube membrane proteins by Western Blotting. SNX17 overexpression significantly increased cell surface LRP4 experssion (1.33 ± 0.06) (**Figure 3B**), along with a significantly higher MuSK phosphorylation level (1.24 ± 0.04) after Agrin stimulation than that in the SNX17 OE-con group (**Figure 3C**). In terms of AChR clustering, when Agrin-alone was applied, SNX17 overexpression increased the number of AChR clusters (54.00 ± 3.79) compared to the SNX17 OE-con group (41.33 ± 2.03), and length of AChR clusters (11.80 ± 0.95 μm) was also longer than that of the SNX17 OE-con group (7.88 ± 0.90 μm). When cells were again treated with a mixed application of Agrin and anti-LRP4 antibody, the number of AChR clusters in the SNX17 OE group (42.33 ± 0.88) was still higher than the SNX17 OE-con group (28.67 ± 0.67) (**Figure 3D**). These results demonstrate the role of SNX17 in promoting MuSK phosphorylation and AChR aggregation by increasing the amount of internalized LRP4 recycled to the membrane.

### *3.3 SNX17* expression decreased in the gastrocnemius muscle in experimental autoimmune MG mice, along with AChR fragmentation at the NMJ

**Figure 4:**
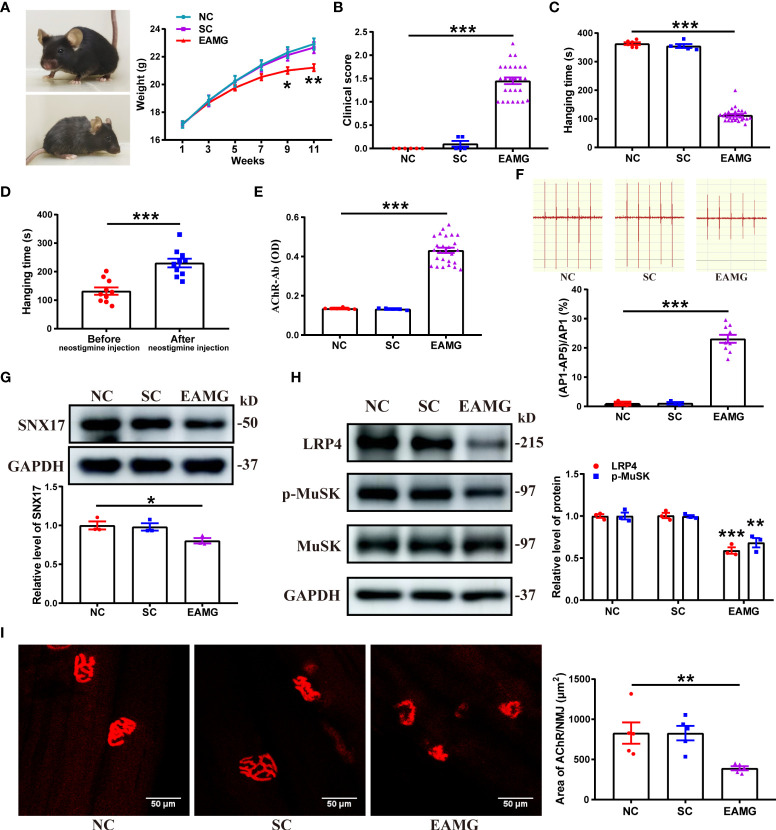
SNX17 expression was decreased in the gastrocnemius muscle of EAMG mice, and AChR was fragmented at the NMJ. **(A)** Representative mice in the normal group (upper left) and the EAMG group (lower left); weight gain in mice in each group between 1 and 11 weeks (right panel). NC: n = 6; SC: n = 5; EAMG: n = 26. **(B)** Lennon grading method to clinical score myasthenic symptoms in mice in each group. NC: n = 6; SC: n = 5; EAMG: n = 26. **(C)** Hanging time of mice in each group was measured by the flip suspension test. NC: n = 6; SC: n = 5; EAMG: n = 26. **(D)** Hanging time of 10 randomly selected EAMG mice before and 20 min after injecting neostigmine, measured by the flip suspension test. n = 10/group. **(E)** Concentration of AChR-Ab in serum was detected using ELISA. NC: n = 6; SC: n = 5; EAMG: n = 26. **(F)** EMG of the gastrocnemius muscle was determined using low-frequency RNS. The attenuation rate of the fifth amplitude of EMG compared with the first amplitude was calculated. NC and SC: n = 3/group; EAMG: n = 10. Western blotting analyses of the expression levels of **(G)** SNX17, and **(H)** LRP4, MuSK, and p-MuSK in the mouse gastrocnemius muscle tissue. n = 3/group. **(I)** AChR (red) at the NMJ of the gastrocnemius filaments was labeled with R-BTX and the area of AChRs was quantified with ImageJ. n = 5/group; Scale bar = 50 μm. All data are presented as mean ± SEM. *P<0.05, **P<0.01, ***P<0.001. AChR, acetylcholine receptor; EAMG, experimental autoimmune myasthenia gravis; LRP4, low-density lipoprotein receptor-related protein 4; MuSK, muscle-specific receptor tyrosine kinase; NC, normal control; NMJ, neuromuscular junction; R-BTX, rhodamine-labeled bungarotoxin; RNS, repetitive nerve stimulation; SC, solvent control; SEM, Standard Error of Mean; SNX17, sorting nexin 17.

Next, we used AChR-α subunit (Rα 97–116) peptides as immunogens to establish EAMG models through active immunization and explore changes in SNX17 in the EAMG models. First, the EAMG model was screened and evaluated, and 26 mice that met the EAMG model criteria were included; the modeling success rate was 65%. Compared to conditions in normal mice, EAMG mice had dull hair, a raised back, decreased activity, and slower weight gain (**Figure 4A**). EAMG mice also had higher clinical scores (**Figure 4B**) and shorter hang times (**Figure 4C**) than control mice, demonstrating poor limb strength. However, inhibition of cholinesterase at the NMJ by injection of neostigmine restored limb strength in EAMG mice with a significant increase in hanging time (**Figure 4D**). In addition, AChR-Ab was positive in the sera of EAMG mice (**Figure 4E**). The fifth amplitude attenuation rate of gastrocnemius EMG was 23.12% ± 1.37% (**Figure 4F**). Second, we analyzed SNX17 expression in the gastrocnemius muscle of EAMG mice. SNX17 expression (0.80 ± 0.03) was lower (**Figure 4G**), and LRP4 protein (0.59 ± 0.04) and MuSK phosphorylation levels (0.68 ± 0.06) were lower (**Figure 4H**) than those in the NC and SC groups. Finally, we observed morphological changes in AChRs at the NMJ on myofilaments and quantified the area of AChRs. AChRs at the NMJ in the NC and SC groups were morphologically normal with a complete pretzel-shaped structure. In contrast, the area of AChRs at the NMJ was significantly reduced in EAMG mice (390.60 ± 25.64 μm^2^) compared with the NC (828.80 ± 133.00 μm^2^) and SC (827.70 ± 90.12 μm^2^) groups, and the AChRs were incomplete and fragmented (**Figure 4I**).

### 3.4 *SNX17* knockdown decreased LRP4 expression and MuSK phosphorylation in the gastrocnemius muscle of EAMG mice and aggravated the degree of AChR fragmentation at the NMJ and leg weakness

**Figure 5:**
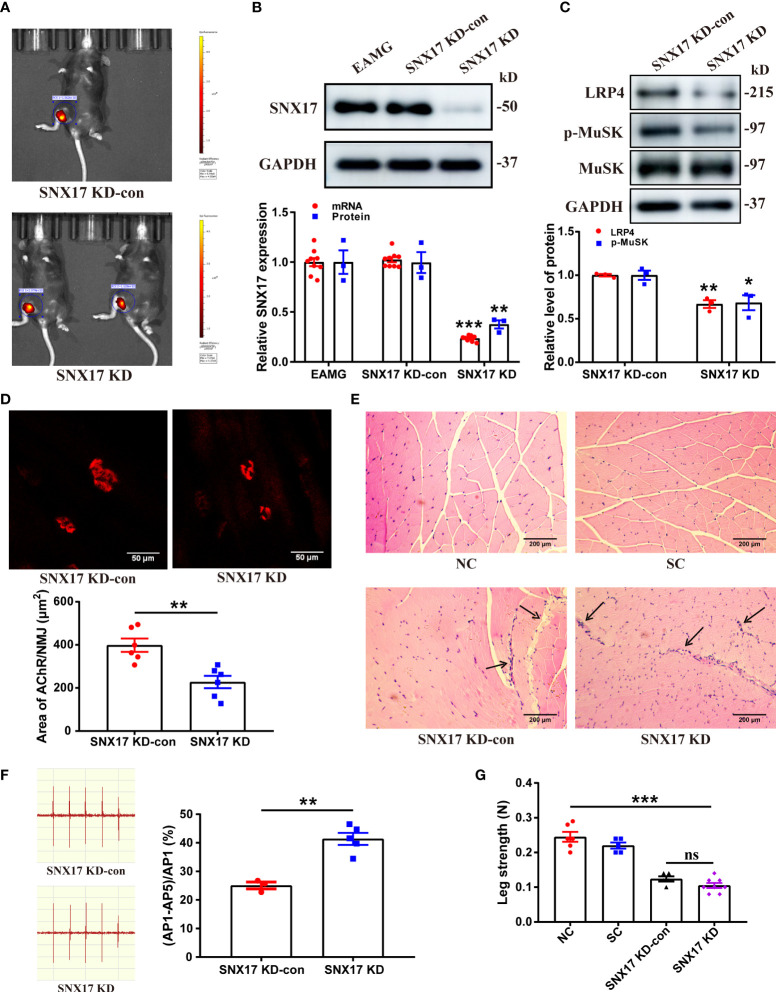
*SNX17* knockdown decreased LRP4 expression and MuSK phosphorylation in the gastrocnemius muscle of EAMG mice and aggravated the degree of AChR fragmentation at the NMJ along with leg weakness. **(A)** Small-animal live imaging of EAMG mice infected with AAV2/9-EGFP NC and AAV2/9-m-SNX17 shRNA-EGFP for 21 days in the gastrocnemius muscle of the right leg. **(B)** Western blotting and qPCR analyses of SNX17 protein (n = 3/group) and mRNA (n = 9/group), respectively, in the mouse gastrocnemius muscle tissue. **(C)** Western blotting analyses of LRP4, MuSK, and p-MuSK levels in the mouse gastrocnemius muscle tissue. n = 3/group. **(D)** AChR (red) at the NMJ of the gastrocnemius filaments was labeled with R-BTX and the area of AChRs was quantified with ImageJ. n = 6/group; Scale bar = 50 μm. **(E)** Hematoxylin and eosin–stained pathological analyses of cross-sections of the gastrocnemius muscle fibers of mice in each group. Black arrows indicate muscle fiber atrophy, rupture, or inflammatory cell infiltration. Scale bar = 200 μm. **(F)** EMG of the gastrocnemius muscle was measured using low-frequency RNS. The attenuation rate of the fifth amplitude of EMG compared with the first amplitude was calculated. SNX17 KD-con: n = 3; SNX17 KD: n = 5. **(G)** Bar dynamometer measurement of the right leg strength of mice in each group. NC: n = 6; SC: n = 5; SNX17 KD-con: n = 5; SNX17 KD: n = 8. All data are presented as mean ± SEM. *P<0.05, **P<0.01, ***P<0.001. AChR, acetylcholine receptor; EAMG, experimental autoimmune myasthenia gravis; LRP4, low-density lipoprotein receptor-related protein 4; MuSK, muscle-specific receptor tyrosine kinase; NC, normal control; NMJ, neuromuscular junction; R-BTX, rhodamine-labeled bungarotoxin; RNS, repetitive nerve stimulation; SC, solvent control; SEM, Standard Error of Mean; SNX17, sorting nexin 17. ns, no significance.

To further explore the effect of SNX17 on AChR at the NMJ in the EAMG model, we injected *SNX17*-knockdown adeno-associated virus into the gastrocnemius muscle of the right leg of EAMG mice; the infection effect was good for 21 days (**Figure 5A**). SNX17 protein and mRNA expression levels were significantly decreased in the gastrocnemius muscle of infected mice compared to the control mice (**Figure 5B**). While *SNX17* knockdown further reduced LRP4 expression (0.67 ± 0.04) and MuSK phosphorylation (0.68 ± 0.09) (**Figure 5C**). The area of AChRs at the NMJ was also further reduced in the SNX17 KD group (227.40 ± 28.64 μm^2^) compared with the SNX17 KD-con group (398.20 ± 30.88 μm^2^), demonstrating intensification in the degree of AChR fragmentation (**Figure 5D**). In addition, we evaluated the internal structure of the gastrocnemius muscle fibers using HE staining. The cross-sections of the muscle fibers in the NC and SC groups were arranged in an orderly manner, and the myocyte nuclei were evenly distributed on the edge of the muscle fibers. In contrast, the muscle fibers in the SNX17 KD-con group atrophied and broken and showed a large amount of inflammatory cell infiltration, whereas the SNX17 KD group also had consistent muscle fiber damage (**Figure 5E**). Compared with the 25.07% ± 1.20% attenuation rate of the fifth amplitude of the gastrocnemius EMG in the SNX17 KD-con group, the attenuation rate of the fifth amplitude of the EMG in the SNX17 KD group increased to 41.38% ± 2.10% (**Figure 5F**). Measurements of unilateral leg strength in mice found that, compared with the SNX17 KD-con group (0.12 ± 0.01 N), the SNX17 KD group (0.11 ± 0.01 N) slightly reduced the leg strength of mice, but the difference was not statistically significant (*P* > 0.05), though it was significantly lower than that of NC group mice (0.25 ± 0.01) (**Figure 5G**).

### 3.5 *SNX17* overexpression increased LRP4 expression and MuSK phosphorylation in the gastrocnemius muscle of EAMG mice and improved AChR morphology at the NMJ and leg weakness

**Figure 6:**
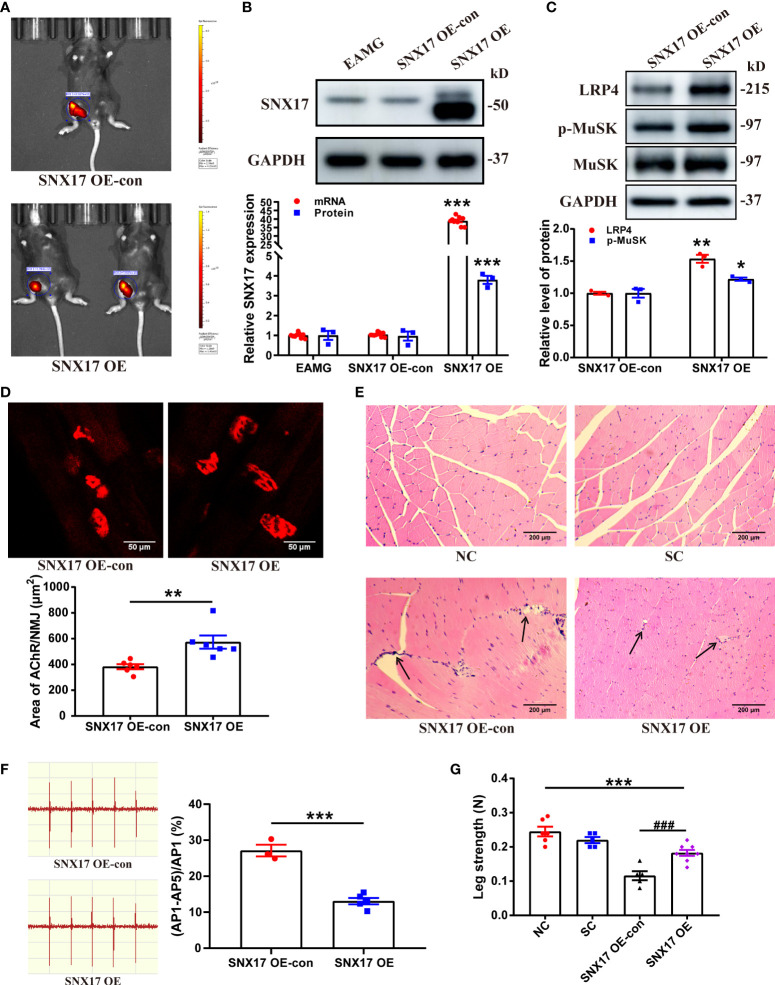
SNX17 overexpression increased LRP4 expression and MuSK phosphorylation in the gastrocnemius muscle of EAMG mice and improved AChR morphology at the NMJ and leg weakness. **(A)** Small-animal live imaging of EAMG mice infected with AAV2/9-ZsGreen and AAV2/9-CMV-m-SNX17-3xflag-ZsGreen for 21 days in the gastrocnemius muscle of the right leg. **(B)** Western blotting and qPCR analyses of SNX17 protein (n = 3/group) and mRNA (n = 9/group) in the gastrocnemius muscle tissue. **(C)** Western blotting analyses of LRP4, MuSK, and p-MuSK expression in the gastrocnemius muscle tissue. n = 3/group. **(D)** AChR (red) at the NMJ of the gastrocnemius filaments was labeled with R-BTX and the area of AChRs was quantified with ImageJ. n = 6/group; Scale bar = 50 μm. **(E)** Hematoxylin and eosin-stained pathological analyses of cross-sections of the gastrocnemius muscle fibers of mice in each group. Black arrows indicate muscle fiber atrophy, rupture, or inflammatory cell infiltration. Scale bar = 200 μm. **(F)** EMG of the gastrocnemius muscle was assessed using low-frequency RNS. The attenuation rate of the fifth amplitude of EMG compared with the first amplitude was calculated. SNX17 OE-con: n = 3; SNX17 OE: n = 5. **(G)** Bar dynamometer measurement of the right leg strength of mice in each group. NC: n = 6; SC: n = 5; SNX17 KD-con: n = 5; SNX17 KD: n = 8. All data are presented as mean ± SEM. *P<0.05, **P<0.01, ***P<0.001, ^###^P<0.001. AChR, acetylcholine receptor; EAMG, experimental autoimmune myasthenia gravis; LRP4, low-density lipoprotein receptor-related protein 4; MuSK, muscle-specific receptor tyrosine kinase; NMJ, neuromuscular junction; R-BTX, rhodamine-labeled bungarotoxin; RNS, repetitive nerve stimulation; SC, solvent control; SEM, Standard Error of Mean; SNX17, sorting nexin 17.

Furthermore, we established an EAMG mouse model that overexpressed *SNX17*; the infection effect was good on the 21st day (**Figure 6A**), along with a significant increase in SNX17 protein and mRNA expression levels in the gastrocnemius muscle tissue (**Figure 6B**). However, the size of overexpressed SNX17 in the western blot band was slightly smaller than that of endogenous SNX17, but the reason for the size difference is unclear. SNX17 overexpression increased LRP4 expression (1.54 ± 0.06) and MuSK phosphorylation levels (1.22 ± 0.03) (**Figure 6C**). Compared with the SNX17 OE-con group (382.90 ± 19.60 μm^2^), the area of AChRs at the NMJ in the SNX17 OE group (573.70 ± 51.29 μm^2^) was significantly increased, the fragmentation degree of AChRs was reduced, and the structure was basically intact (**Figure 6D**). In addition, in the gastrocnemius muscle fibers of *SNX17*-overexpressing mice, no obvious damage phenomena such as inflammatory cell infiltration and muscle fiber atrophy were observed (**Figure 6E**). Compared with the 27.12% ± 1.63% attenuation rate of the fifth amplitude of the EMG in the SNX17 OE-con group, the attenuation rate of the fifth amplitude of the EMG in the SNX17 OE group was reduced to 13.07% ± 0.89% (**Figure 6F**). Although the leg strength of SNX17-overexpressing mice (0.18 ± 0.01 N) was still different from that of the NC group mice (0.25 ± 0.01 N), it also recovered to a certain extent compared with the SNX17 OE-con group (0.12 ± 0.01 N) (**Figure 6G**).

## 4 Discussion

SNX17 is an intracellular adaptor protein that regulates the degradation and recycling of internalized membrane proteins and exerts its “sorting” function. SNX17 function is known to play an important role in various diseases (21–23), but whether it also plays a role in MG has not yet been elucidated. The novel findings of this study confirmed the intracellular interaction between SNX17 and LRP4, a key protein in the pathogenesis of MG. At the cellular level, we validated that SNX17 increased cell surface LRP4 experssion to promote MuSK phosphorylation and AChR aggregation. In the EAMG model, the content of SNX17 molecules in the gastrocnemius muscle was reduced, and the morphology of AChR at the NMJ was incomplete, showing obvious fragmentation. Upregulation of SNX17 in the gastrocnemius muscle of EAMG mice can increase the LRP4 content in the endplate membrane, significantly improve the fragmented morphology of AChR at the NMJ, alleviate the pathological damage of gastrocnemius muscle fibers, and the leg weakness symptoms of EAMG mice are recovered to a certain extent. Our findings demonstrate the effective role of SNX17 on muscle strength recovery in MG, and it can be used as a potentially important target for another symptomatic drug therapy besides pyridostigmine bromide which is the classic symptomatic treatment for MG.

The exact mechanism by which SNX17 regulates the recycling of internalized membrane proteins into the membrane has been well studied. SNX17 precisely recognizes the NPxY/NxxY motif in internalized membrane proteins through its FERM domain and binds to internalized membrane proteins. SNX17 simultaneously associates with the “retriever” complex and couples with the CCC and WASH complexes, thereby preventing the bound, internalized membrane protein from entering the lysosome for degradation and promoting recycling to the cell surface (24). In this study, SNX17 significantly increased the cell surface LRP4 experssion, which might be related to SNX17 promoting the recycling of internalized LRP4 back to the membrane. However, whether SNX17 mediates the recycling of LRP4 to increase cell surface LRP4 expression as per the aforementioned mechanism remains to be confirmed and warrants further research.

As a type I single-pass transmembrane protein, LRP4 easily binds to its ligands and mediates endocytosis (25), and is necessary for the formation and development of NMJ synapses (26, 27). The Agrin–LRP4–MuSK signaling pathway involved and mediated by LRP4 is one of the important mechanisms for inducing AChR cluster aggregation (28). Studies have shown that Agrin secreted from motor neuron fiber terminals binds to LRP4 on the NMJ muscle cell membrane, induces MuSK phosphorylation, and promotes the aggregation of AChR at the postsynaptic membrane (29). This study was based on the induction of AChR cluster aggregation by LRP4 and found and confirmed that overexpression of SNX17 can promote AChR clustering.

The stabilization of aggregated AChRs at the NMJ postsynaptic membrane is critical for neuromuscular excitation transmission (30). In the EAMG model of this study, the AChR at the NMJ was significantly fragmented. However, impaired AChR aggregation cannot enable better binding of ACh released from the presynaptic membrane, which hinders the transmission of NMJ excitatory signals and may cause muscle fiber damage, and ultimately leading to the occurrence of NMJ-like diseases including MG (31, 32). In this study, by overexpressing SNX17 in the gastrocnemius muscle of EAMG mice to increase the content of LRP4, the above situation was effectively reversed, and the symptoms of muscle weakness were significantly relieved. Therefore, maintaining the stability of LRP4 on the NMJ muscle cell membrane is not only critical for the normal aggregation of AChR clusters at the postsynaptic membrane and recovery of NMJ structure and function but may also be another viable avenue for the treatment of MG (33). However, clinical studies have shown that among MG patients, 85% are anti-AChR antibody-positive MG (AChR-MG), 6% are anti-MuSK antibody-positive MG (MuSK-MG) (34), and 2% are LRP4-MG (35, 36). Because AChR-MG patients account for the largest proportion in MG patients, promoting normal aggregation of AChR in MG is key in ensuring the normal transmission of neuromuscular excitation and restore muscle contraction function in MG patients (2). Therefore, in the animal model of this study, we first explored the effects of SNX17 on AChR aggregation in the AChR-MG mouse model. Although our study confirmed the beneficial role of SNX17 in AChR-MG, its role in MuSK-MG, LRP4-MG remains to be further explored. In addition, the evaluation of SNX17 in this study was mainly in the gastrocnemius muscle, and the MG crisis mainly causes weakness of the respiratory muscles (e.g., intercostal muscles, diaphragm) (37). Therefore, expanding the exploration of the role of SNX17 in respiratory muscles in the future may help find a novel method to treat the weakness of patients in crisis.

**Figure 7:**
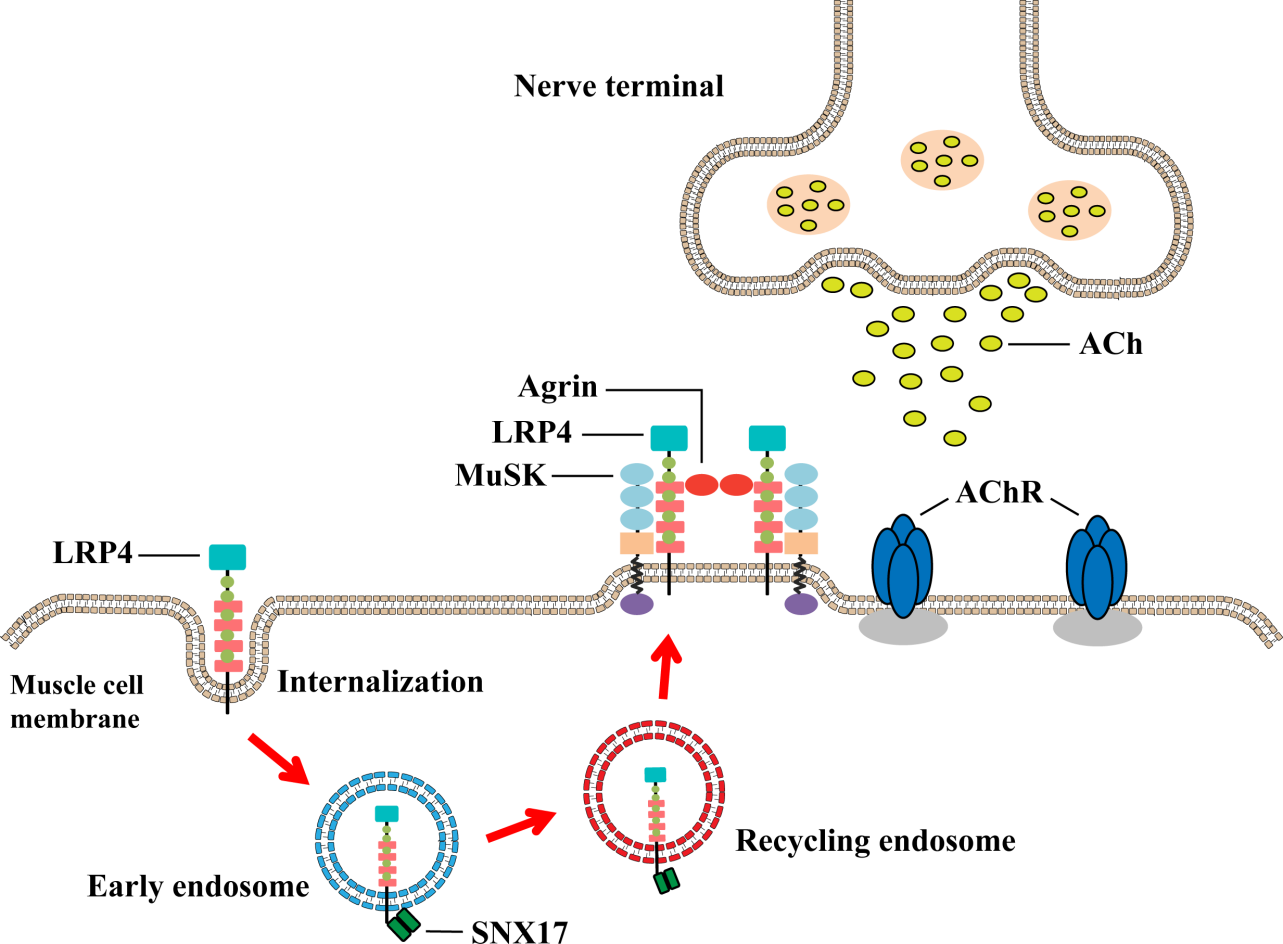
Underlying mechanism by which SNX17 increases cell surface LRP4 expression to promote AChR aggregation and neuromuscular excitatory transmission restoration. SNX17 recognizes and binds LRP4 that is internalized into early endosomes and mediates the transformation of LRP4 to circulating endosomes and then back to the muscle cell membrane. LRP4 returning to the membrane induces AChR aggregation through the Agrin–LRP4–MuSK signaling pathway, which binds ACh released from the presynaptic membrane and further restores the neuromuscular excitation transmission. AChR, acetylcholine receptor; LRP4, low-density lipoprotein receptor-related protein 4; MuSK, muscle-specific receptor tyrosine kinase; NMJ, neuromuscular junction; SNX17, sorting nexin 17.

Taken together, this study elucidated the mechanism by which SNX17 ameliorates muscle weakness in EAMG mice by interacting with internalized LRP4 to increase cell surface LRP4 expression to promote MuSK phosphorylation and AChR aggregation (**Figure 7**). This new mechanism is useful for supporting and complementing the involvement of SNX17 in receptor recycling and suggests that SNX17 may be a new target for future MG therapy.

## 5 Conclusions

SNX17 promotes MuSK phosphorylation and AChR aggregation by increasing cell surface LRP4 expression. Based on the above mechanism, upregulation of SNX17 in the gastrocnemius muscle of EAMG mice can significantly improve the fragmented morphology of AChR at the NMJ, alleviate the pathological damage of muscle fibers and restore leg muscle weakness to a certain extent, revealing that SNX17 may be a future MG therapy new target.

## Data availability statement

The original contributions presented in the study are included in the article. Further inquiries can be directed to the corresponding author.

## Ethics statement

The animal study was reviewed and approved by The Laboratory Animal Management and Ethics Committee of the Henan Institute of Medical and Pharmaceutical Sciences.

## Author contributions

FG conceived the project. XH conducted cell experiments with the assistance of YJ, YiZ, and JL. XH conducted animal experiments with the assistance of SZ, SQ, JZ, and XZ. WC, WL, PL, and LZ performed data analysis. TS, HF, JY, YuZ, XC, and QZ provided technical assistance. XH prepared the manuscript and had discussion with the other authors. All authors contributed to the article and approved the submitted version.

## Funding

This work was supported by Project of Basic Research Fund of Henan Institute of Medical and Pharmacological Sciences (grant numbers 2022BP0102, 2020BP0201, 2022BP0117), special project of Henan Province of Traditional Chinese Medicine scientific research (grant number 2021ZY1044), Henan Province scientific and technological research (grant number 212102310153) and Zhongyuan science and technology innovation leading talent project (grant number 224200510027).

## Acknowledgments

We are grateful for the guarantee and support provided by the Henan Engineering Technology Research Center for Accurate Diagnosis Neuroimmunity and Key Laboratory of Pharmacology for Liver Diseases of Henan Province for the smooth implementation of various experiments in this study.

## Conflict of interest

The authors declare that the research was conducted in the absence of any commercial or financial relationships that could be construed as a potential conflict of interest.

## Publisher’s note

All claims expressed in this article are solely those of the authors and do not necessarily represent those of their affiliated organizations, or those of the publisher, the editors and the reviewers. Any product that may be evaluated in this article, or claim that may be made by its manufacturer, is not guaranteed or endorsed by the publisher.

## Abbreviations

AChR, acetylcholine receptor; Con, control; CFA, complete Freund’s adjuvant; EAMG, experimental autoimmune myasthenia gravis; IFA, incomplete Freund’s adjuvant; KD, knockdown; LDLR, low-density lipoprotein; LRP4, low-density lipoprotein receptor-related protein 4; MuSK, muscle-specific receptor tyrosine kinase; MG, myasthenia gravis; NC, normal control; NMJ, neuromuscular junction; PBS, phosphate-buffered saline; PX, Phox-homology; OE, overexpression; R-BTX, rhodamine-labeled bungarotoxin; RIPA, radioimmunoprecipitation assay; RNS, repetitive nerve stimulation; SC, solvent control; SPF, specific pathogen-free; SNX17, sorting nexin 17.
